# Metabolomic analysis shows differential hepatic effects of T_2_ and T_3_ in rats after short-term feeding with high fat diet

**DOI:** 10.1038/s41598-017-02205-1

**Published:** 2017-05-17

**Authors:** Liliana F. Iannucci, Federica Cioffi, Rosalba Senese, Fernando Goglia, Antonia Lanni, Paul M. Yen, Rohit A. Sinha

**Affiliations:** 1 Program of Cardiovascular and Metabolic Disorders, Duke-NUS Medical School Singapore8 College Road, 169857 Singapore, Singapore; 2 Dipartimento di Scienze e Tecnologie, Università degli Studi del Sannio, Benevento, Italy; 3 Dipartimento di Scienze e Tecnologie Ambientali, Biologiche e Farmaceutiche, Seconda Università degli Studi della Campania “Luigi Vanvitelli” Napoli, Caserta, Italy

## Abstract

Nonalcoholic fatty liver disease (NAFLD) is a major health problem worldwide, and is often associated with lipotoxic injury, defective mitochondrial function, and insulin resistance. Thyroid hormones (THs) are important regulators of hepatic lipid metabolism. Among the THs, diiodothyronine (T_2_) and triiodothyronine (T_3_) have shown promising results in lowering hepatic fat content in various models of NAFLD. In this study, we used a targeted metabolomics approach to investigate the differential effects of T_2_ and T_3_ on the early metabolic adaptation in the livers of rats fed high fat diet (HFD), a period when hepatosteatosis is reversible. Our results showed that both T_2_ and T_3_ strongly induced autophagy and intra-hepatic acylcarnitine flux but prevented the generation of sphingolipid/ceramides in animals fed HFD. Interestingly, although both T_2_ and T_3_ decreased hepatic fat content, only T_2_ was able to rescue the impairment in AKT and MAPK/ERK pathways caused by HFD. In summary, we have identified and characterized the effects of T_2_ and T_3_ on hepatic metabolism during short-term exposure to HFD. These findings illuminate the common and divergent metabolic pathways by T_2_ and T_3_ that also may be important in the prevention and treatment of NAFLD.

## Introduction

Non-alcoholic fatty liver disease (NAFLD) is a major health problem associated with obesity and diabetes. It affects more than 40% of the U.S. population and currently is the single largest cause of liver transplants worldwide^1^. NAFLD is a spectrum of diseases that starts with hepatosteatosis and can progress to non-alcoholic steatohepatitis (NASH) that eventuates in cirrhosis. The molecular mechanism(s) for NAFLD and its progression still are not fully understood and currently there are no approved drug therapies for NAFLD^2^. Recent studies suggest that NAFLD is not only due to excess triglyceride storage but also to the accumulation of other lipid species that potentially are cytotoxic and induce inflammation in hepatocytes^3^. Cytotoxic lipid species such as ceramides can induce mitochondrial dysfunction resulting in enhanced Reactive oxygen species (ROS) production, which is thought to represent the central abnormality responsible for the progression from simple fatty liver to NASH and impaired hepatic insulin action^4, 5^. These changes are associated with other metabolic features such as defective amino acid metabolism, impaired TCA cycle flux, and unregulated hepatic glucose production^6^.

Thyroid hormones (THs) and their analogues have long been recognized as important regulators of hepatic lipid metabolism, and studies in rodents suggest that they can reduce hepatosteatosis^7^. TH actions were thought to be classically mediated by 3,3′5-triiodothyronine (T_3_) and levothyroxine (T_4_); however, in recent years, another TH-related compound, 3,5-diiodothyronine (T_2_), has been shown to be bioactive. Both T_3_ and T_2_ can influence several aspects of energy expenditure^8^. In particular, one of the prominent characteristics of TH signaling is a rapid induction in increasing oxygen consumption and ATP production. Recent studies also have linked this signaling pathway to fatty acid oxidation (FAO) in a variety of tissues^9, 10^. Although T_3_ has long been thought to exert its effects primarily by binding to nuclear TH receptors (TRs)^11, 12^, the mechanism of action for T_2_ still remains not entirely clarified even if some mechanisms have been proposed such as the activation of sirtuin1 (SIRT1)^13^ and a direct interaction with the subunit Va of the cytochrome oxidase in bovine heart^14, 15^. Initial reports described T_2_ showing a difference in the non-genomic effects on oxygen consumption when compared to those induced by T_3_^16^. Subsequent studies showed that T_2_ also is able to act at the genomic level and modulate gene expression, suggesting that it could be an alternative ligand for TRβ1^16^.

Metabolomics is a novel technology that has emerged as a powerful tool for identifying metabolite biomarkers associated with NAFLD pathogenesis. Metabolomics provides a comprehensive view of the changes in several metabolic and signaling pathways and their interactions^17–23^. In this connection, THs such as T_3_ and T_2_ have been shown to reduce hepatic fat accumulation in both animal and cell culture models^7, 24–28^. However, a comprehensive and comparative metabolomic analysis of T_2_ and T_3_ is lacking. Most of our current knowledge of the actions of T_2_ and T_3_ were demonstrated independently without a side-by-side comparison of their effects on hepatic metabolism. Additionally, our understanding of the events associated with the early stages of NAFLD is very limited since the focus of the field has been on the diet-induced or genetic models of chronic obesity and NAFLD. The early adaptive stages of NAFLD are important since they may not only help understand the metabolic changes that lead to progression of NAFLD, but also identify potential drug targets for the treatment of hepatosteatosis when it still is reversible. In this paper, we focused on the relative effects of T_2_ and T_3_ in hepatic adaptation to acute feeding of high fat diet in rats. For this purpose, we sought to gain a comprehensive metabolic view, analyzing different hepatic metabolites related to lipid, ceramide, and amino acid metabolism in response to short-term HFD and its modulation by T_2_ or T_3_ treatment. Our results provide novel insights into the differential effects of T_2_ and T_3_ on hepatic lipid metabolism, mitochondrial function, and insulin signaling pathways after a short-term HFD regimen.

## Results

### T_2_ and T_3_ effects on hepatic TG accumulation and acylcarnitines in rats fed HFD

**Figure 1:**
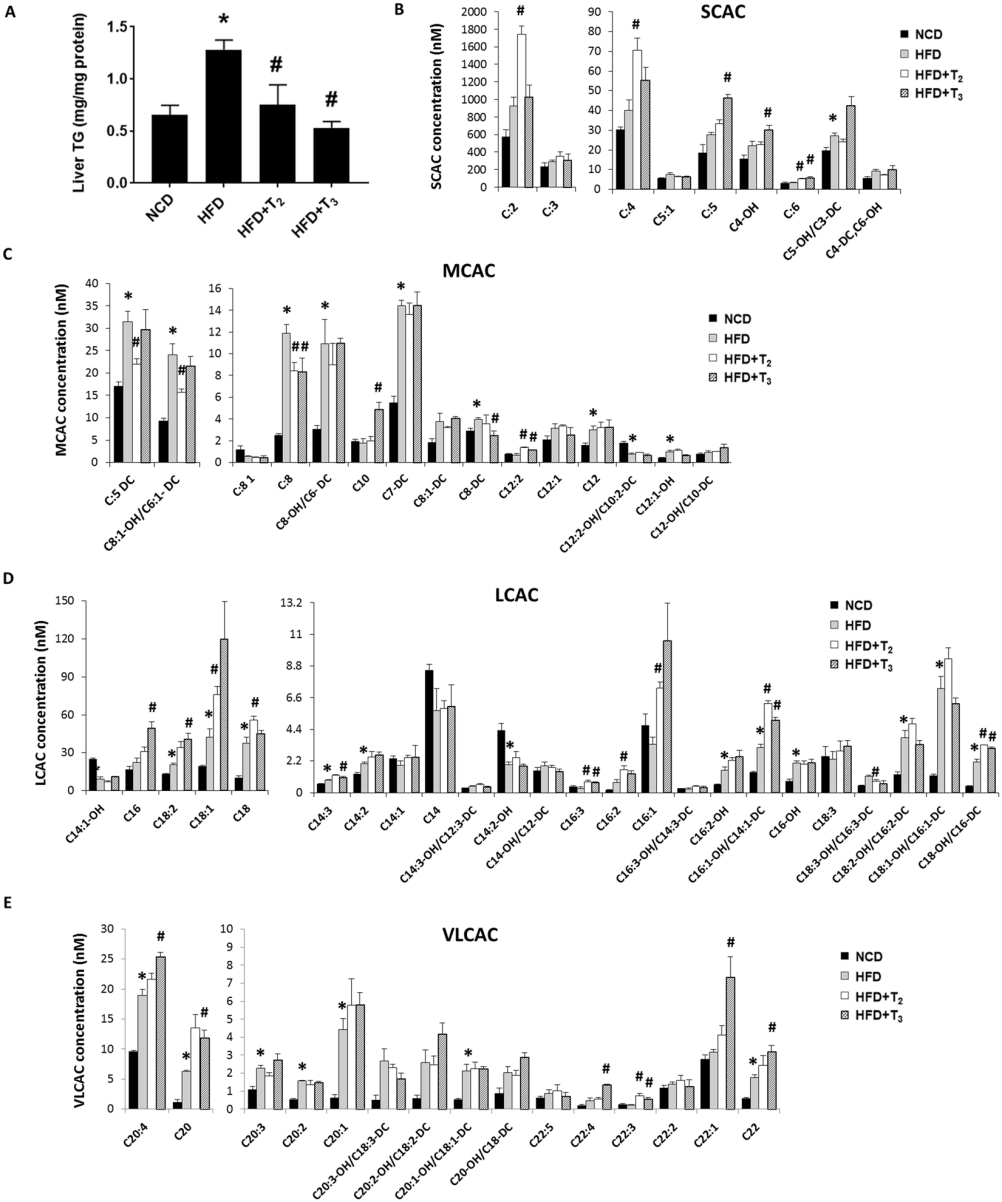
T_2_ and T_3_ reduce hepatic lipid accumulation in HFD fed rats associated with increased acylcarnitine flux. (**A**) Representative graph showing Triglycerides (TGs) content in rats liver treated respectively with NCD, HFD, HFD+T_2_, HFD+T_3_ for 1 week. Values are means ± SEM (n = 4). *P < 0.05 in NCD Vs HFD; ^#^P < 0.05 in HFD Vs HFD+T_2_/HFD+T_3_. Metabolomics profiles of (**B**) short chain acylcarnitines (SCAC), (**C**) medium chain acylcarnitines (MCAC), (**D**) long chain acylcarnitines (LCAC) and (**E**) very long chain acylcarnitines (VLCAC) in NCD, HFD, HFD+T_2_ and HFD+T_3_ treated rats. Values are means ± SEM (n = 4). *P < 0.05 in NCD Vs HFD; ^#^P < 0.05 in HFD Vs HFD+T_2_/HFD+T_3_.

Previous studies showed that T_2_ and T_3_ influenced hepatic lipid metabolism by virtue of their abilities to stimulate fatty acid β-oxidation (FAO) both *in vitro* and *in vivo* ^16, 29^. Thus, we sought to characterize the primary effect of T_2_ and T_3_ on lipid catabolism through analysis of β-oxidative intermediates in hepatic tissues. Accordingly, we first measured hepatic triglyceride (TG) levels and found a significant decrease in both T_2_- and T_3_-treated rats fed HFD *vs*. rats fed HFD alone (Fig. 1), consistent with earlier reports^25–28^. The body weight, serum iodothyronine, and blood glucose levels of animals in each group are provided (Suppl Fig. 1).

We next performed metabolomic profiling of hepatic acylcarnitines species ranging in size from 2 to 22 carbons. Acylcarnitine esters are synthesized from their respective acyl-CoA intermediates by enzymes known as carnitine acyltransferases residing in mitochondria to promote their import into mitochondria whereupon they undergo FAO. Long chain fatty acids represent substrates derived from lipolysis of triglycerides from adipose tissue, hepatic stores, and diet. Even chain acylcarnitine species from C6 to C22 represent fatty acid metabolites due to incomplete fatty acid oxidation. Odd short-chain species, such as C3 and C5 are mostly formed by amino acid catabolism whereas C4 is derived from both fatty acids and amino acids. Acylcarnitine C2, which is used as a proxy measure of acetyl-CoA, is generated by catabolism of fatty acids, amino acids, and/or glucose^30^. C4-OH (hydroxybutyrate) is the end product of fatty acid β-oxidation and also has been used as a marker for this process.

Hepatic acylcarnitine profiling showed that T_2_ and T_3_ had different effects on lipid metabolism in livers from rats fed HFD. We observed that T_2_- and T_3_-treated rats fed HFD displayed increased levels of hepatic short-chain acylcarnitines (SCAC), that are end products from β-oxidation of fatty acids and amino acids as well as from TCA cycle intermediates supplied by amino acids during anapleurosis (Fig. 1B). In this connection, the increase in hepatic C3, C4 and C5 SCACs in T_2_- and T_3_- treated rats occurred in parallel with a decrease in hepatic amino acids levels (Suppl Fig. 2). Our metabolomic analysis also revealed that HFD significantly increased intrahepatic levels of branched-chain amino acid valine (Suppl Fig. 2), which was abolished by T_2_ or T_3_ treatment (Suppl Fig. 2). Similar to the trend observed for valine, other amino acids such as alanine, aspartate, methionine, glutamine, and tryptophan also were increased in livers of rats fed HFD. Furthermore, this effect was reduced by T_2_ or T_3_ treatment, with significant decreases observed by T_2_ for alanine, methionine, glutamine and tryptophan (Suppl Fig. 2). In addition to SCACs, hepatic long-chain and very long-chain acylcarnitines (LCAC, VLCAC) tended to be increased in rats fed HFD, and increased even more in T_2_- and T_3_-treated rats, most likely due to hydrolysis of stored triglycerides as well as influx of FFAs supplied to the liver by lipolysis from adipose tissue (Fig. 1C,D). Medium-chain acylcarnitines (MCAC) also were increased by HFD; however, in contrast to SCACs and LCACs, they were mostly decreased by the two iodothyronines (Fig. 1B). This decrease in MCAC levels compared to the acylcarnitines of other lengths suggested that there might be a relative increase in MCAC flux in the β-oxidation pathway of the liver during treatment by T_2_ or T_3_.

### T_2_ and T_3_ regulation of lipolysis, autophagy, FAO, mitochondrial biogenesis and anti-oxidant proteins in rats fed HFD

**Figure 2:**
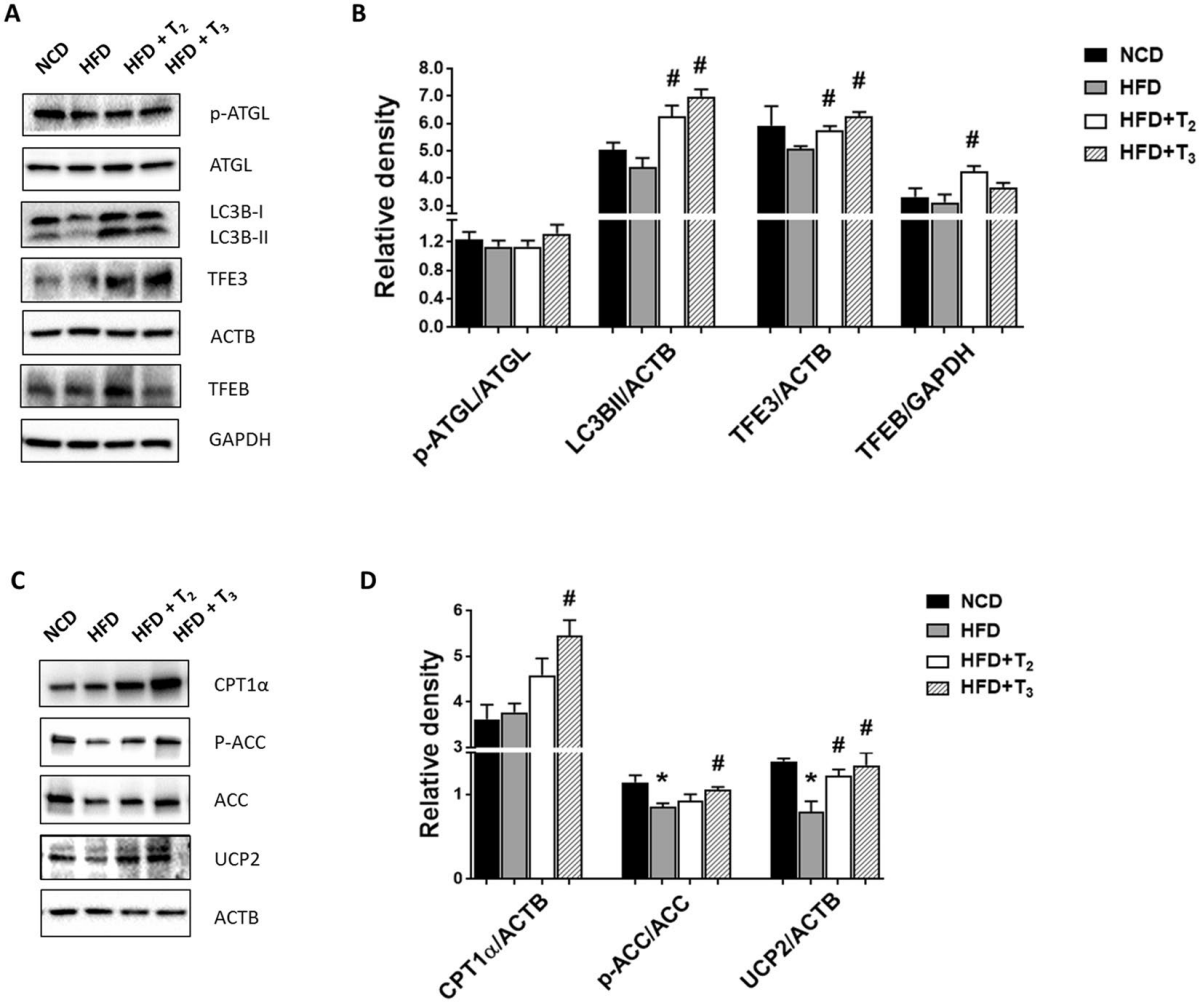
Administration of T_2_ and T_3_ increase lipophagy and FAO regulatory proteins in HFD fed rats. Representative cropped Immunoblots and densitometry showing proteins content of lipolytic and autophagic markers (**A**,**B**), and β-oxidative markers (**C**,**D**). Values are means ± SEM (n = 4). *P < 0.05 in NCD Vs HFD; ^#^P < 0.05 in HFD Vs HFD+T_2_/HFD+T_3_.

To further understand the increases in LCAC and VLAC levels after T_2_ or T_3_ treatment, we assessed the role of the THs on intrahepatic lipolysis. Hepatic TG breakdown is mediated by neutral extralysosomal lipases such as Adipose triglyceride lipase (ATGL)^31^ or via lipophagy, a form of autophagy in which lipids are sequestered into autophagosomes and degraded after autophagosomal fusion with lysosomes via lysosomal acid lipases^32^. Our results demonstrated that both T_2_ and T_3_ had no significant effects on ATGL phosphorylation, a marker for ATGL activity (Fig. 2A,B). In contrast, we found that both T_2_ and T_3_ induced autophagy proteins such as Microtubule-associated protein 1A/1B-light chain 3 B-II (LC3B-II), and Transcription factor E3 (TFE3) expression (Fig. 2A,B). T_2_ also increased Transcription factor EB (TFEB) expression significantly but T_3_ did not. These results suggested that the increases in lipolysis by both T_2_ and T_3_ were likely mediated by autophagy/lipophagy rather than by ATGL activity.

We next examined the presence of mitochondrial β-oxidation markers in liver samples from rats fed HFD and treated with T_2_ or T_3_ (Fig. 2C,D). T_3_ treatment significantly increased Carnitine palmitoyltransferase I alpha (CPT1α) protein expression and T_2_ tended to increase CPT1α protein expression in mice fed HFD although not significant (Fig. 2C,D). Moreover, HFD decreased levels of Uncoupling protein 2 (UCP2) whereas both T_2_ and T_3_ treatments reversed these effects (Fig. 2C,D). Acetyl-CoA carboxylase (ACC) phosphorylation increases FAO in hepatic cells.Interestingly, only T_3_ was able to significantly rescue the decrease in p-ACC/ACC levels induced by HFD, with T_2_ having only a minor effect (Fig. 2C,D). These data suggested that in general both T_2_ and T_3_ increased the levels of pro-FAO proteins to assist entry of fatty Acyl-CoAs into the mitochondria leading to increased FAO and oxidative phosphorylation. Taken together these data suggested that both iodothyronines increased hydrolysis of hepatic triglycerides and FAO to prevent the development of fatty liver. We also observed that that only T_3_ significantly increased expression of mitochondrial biogenesis markers such as Peroxisome proliferator-activated receptor gamma coactivator 1-alpha (PGC1α), Nuclear respiratory factor 1(NRF-1), and Transcription factor A, mitochondrial (TFAM) (p = 0.06) in livers from rats fed HFD (Suppl Fig. 3A,B). Surprisingly, T_2_ had no effect on these markers. Furthermore, HFD induced the expression of the anti-oxidant enzyme, Superoxide dismutase 1(SOD1) (Suppl Fig. 4). T_3_ decreased SOD1 protein expression in livers from rats fed HFD whereas T_2_ maintained the same level as HFD alone (Suppl Fig. 4). Taken together, these data suggest that T_3_ and T_2_ may utilize different mechanisms to handle the increased ROS and oxidized mitochondrial proteins due to increased oxidative phosphorylation.

### T_2_ and T_3_ effects on the induction of hepatic sphingolipid synthesis in rats fed HFD

**Figure 3:**
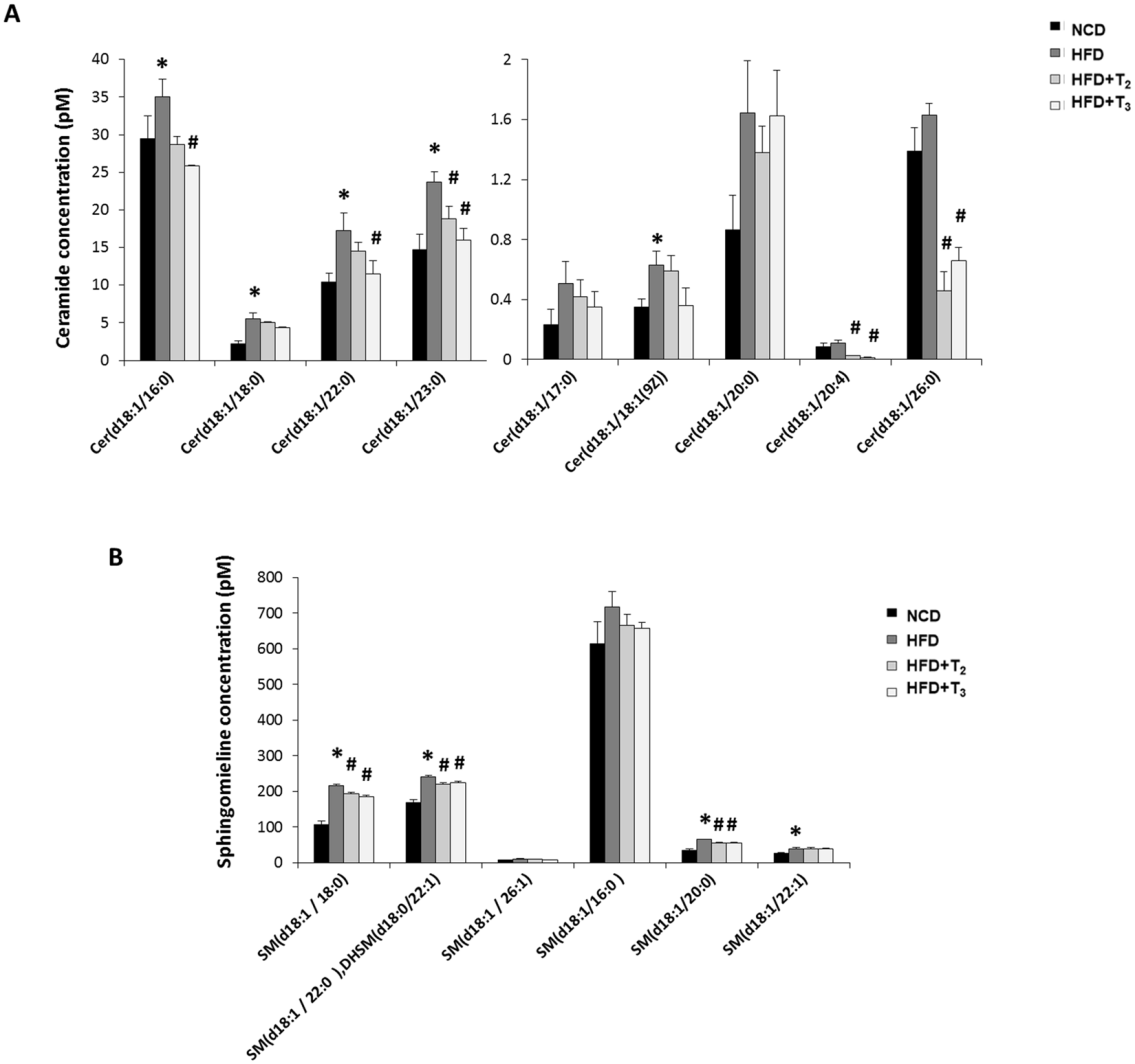
HFD-induced Sphingolipids accumulation is prevented by T_2_ and T_3_ treatment. Quantitative analysis of Ceramide (**A**), Sphingomylines and (**B**) Sphinganine after HFD and HFD+T_2_/HFD+T_3_ treatment. Values are means ± SEM (n = 4). *P < 0.05 in NCD Vs HFD; ^#^P < 0.05 in HFD Vs HFD+T_2_/HFD+T_3_.

A diet rich in saturated lipids leads to increased accumulation of cytotoxic lipid species such as the sphingolipid, ceramide and its metabolites. It now is well established that ceramides not only are key structural components of cellular membranes but also important sphingolipid second messengers involved in cellular stress responses^5^. Our metabolomic profiling showed a consistent increase in almost all the examined hepatic ceramide species from the livers of rats fed HFD compared to rats fed NCD (Fig. 3A). Interestingly, we found that rats treated with either of the two iodothyronines rescued the HFD effect by decreasing the levels of most of the ceramide species (Fig. 3A). Other sphingolipids such as sphingomyelin were stimulated by HFD but were reduced after T_2_ or T_3_ treatment (Fig. 3B).

### Selective T_2_ and T_3_ effects on MAPK and PI3K signaling in livers of rats fed HFD

**Figure 4:**
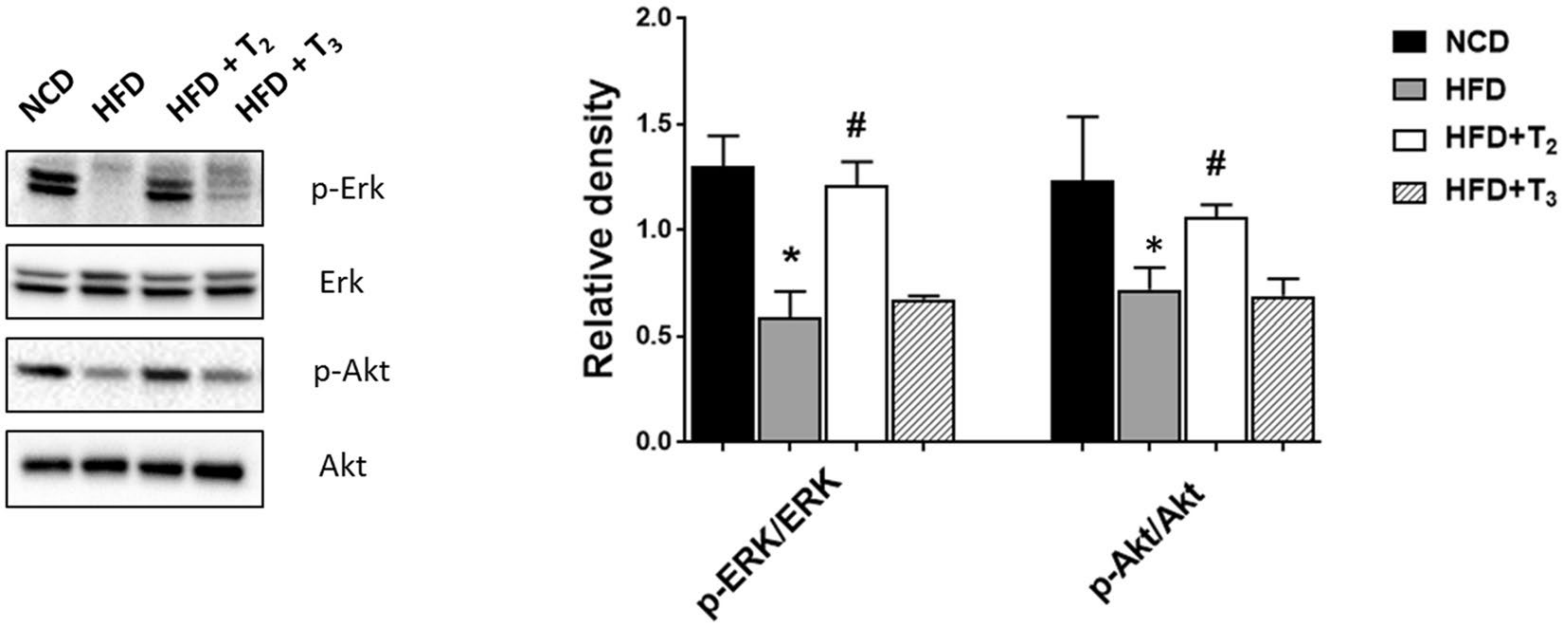
T_2_ but not T_3_ affects insulin/growth factor signaling in livers of HFD fed rats. Representative cropped Immunoblot and densitometry of ERK and AKT protein levels showing THs influence on the correlated signaling pathway. Values are means ± SEM (n = 4). *P < 0.05 in NCD Vs HFD; ^#^P < 0.05 in HFD Vs HFD+T_2_/HFD+T_3_.

HFD is associated with impaired insulin signaling in both animals and humans^33^. This effect generally is attributed to increased TG accumulation, ceramide signaling, and accumulation of branched-chain amino acids^4, 34^. Since we observed a protective effect of T_2_ and T_3_ on these parameters, we next investigated their effects on insulin/growth factor signaling effectors such as MAPK and PI3K. Taking phosphorylation of ERK and AKT as a marker for active MAPK and PI3K signaling we found that only T_2_ significantly increased ERK and AKT activity whereas T_3_ did not affect these proteins (Fig. 4A,B). These results, therefore, suggested that T_2_ had a very distinct effect on ERK and AKT signaling by T_2_ that was not mediated by T_3_. Interestingly, we observed a significant decrease in the level of TRβ1 in HFD fed rats and this effect was not rescued by either T_2_ or T_3_ (Suppl Fig. 5). These findings raise the possibility that some of the metabolic effects of both T2 and T3 during the adaptive phase may not require TRβ1.

## Discussion

Iodothyronines have become attractive pharmacological compounds to treat NAFLD. Presently, two iodothyronines, T_2_ and T_3_, both have shown efficacy in reducing the severity of NAFLD in both cell culture and animal models of NAFLD^7, 25–28^. It has been proposed that T_2_ and T_3_ employ different mechanisms for their effects, with T_3_ acting at a genomic level and T_2_ via non-genomic signaling^12, 16^. Most previous studies in the literature have not made a side-by-side comparison of the activities of these two iodothyronines; thus, our understanding of the relative differences between both their biological and metabolic effects is limited. In this study, we used a state-of-the-art metabolomics approach to compare the effects of T_2_ and T_3_ on hepatic adaptation to short-term HFD feeding in rats. The salient findings of our study were: i) Both T_2_ and T_3_ prevented hepatic fat accumulation which was associated with increased autophagy, lipolysis and FAO; ii) T_2_ and T_3_ decreased the synthesis of hepatic sphingolipids such as ceramides in response to HFD; and iii) T_2_, but not T_3_, rescued the impairment of hepatic AKT & ERK signaling pathways in rats fed HFD.

Our results were consistent with earlier reports showing lipid-lowering effects of T_2_ and T_3_ in animal models of NAFLD^7, 25–28^. However, in this study, we employed a more comprehensive metabolomics approach to understand the effects of these iodothyronines on the metabolic processes that control hepatic FAO in rats fed HFD. The increase in acylcarnitines (AC) is representative of increased lipolysis (long-chain AC) as well as increased fatty acid oxidation in the mitochondria (short-chain AC). Hepatic cells exhibit increased AC when fed HFD, due to increased uptake and utilization of fatty acids from HFD. However, the further increase in AC species by both T2 and T3 beyond that observed during HFD feeding alone suggested the induction of lipolysis of hepatic fat stores and fatty acid oxidation, and accounted for the decreased hepatic TG content observed in Fig. 1A. Consistently, we found that SCACs, which serve as proxy markers of FAO, were increased during HFD due to free fatty acids from lipolysis and diet, and further increased after T_2_ and T_3_ treatment due to induction of autophagy/lipophagy, CPT1α expression, and FAO. However, the effects of T_2_ and T_3_ were not uniform across the different SCACs as T_2_ effects were more pronounced on C:2 and C:4 whereas T_3_ caused significant increases in the C:5 and C4-OH (ketogenesis markers) species. These results suggested that although iodothyronines in general increased FAO, T_2_ and T_3_ affected different enzymatic pathways of downstream fatty acid metabolism. Furthermore, since some SCACs are derived from both organic and amino acid oxidation, our data implied that there may be more complex effects of T_2_ and T_3_ on cellular metabolism. Branched-chain amino acids (BCAA) including leucine, isoleucine and valine have been shown to be increased in human NAFLD and positively correlated with hepatic insulin resistance^34^. In this connection, our metabolomic analysis also showed that even short-term feeding on HFD caused a significant increase in the BCAA, valine. Interestingly, both T_2_ and T_3_ were able to rescue this effect. Other amino acids such as phenylalanine, tryptophan, tyrosine and leucine, which are ketogenic in nature, also were decreased by T_2_, and to a lesser extent by T_3_. This down-regulation may be a reflection of the increased oxidation of these amino acids since SCAC levels were increased.

Surprisingly, the levels of MCACs, for the most part, were either suppressed or unchanged by T_2_ and T_3_ treatment when compared with hepatic levels of MCACs from livers of rats fed only HFD. Furthermore, levels of most LCACs and VLCACs were increased in livers from rats fed HFD with T_2_ or T_3_ treatment than those from rats fed HFD alone. This scenario resembles a block in LCAC oxidation; however, when observed in the context of decreased levels of hepatic SCACs from livers from T_2_- and T_3_-treated rats, it is more likely that the reduced MCAC levels were due to increased oxidation of MCACs to SCACs. Furthermore, proteins that were positively-associated with increased FAO such as CPT1α and UCP2 also were increased by T_3_, and to a lesser extent, by T_2_. Finally, since LCACs and VLACs are derived from TG hydrolysis, our results implied that both T_2_ and T_3_ increased lipolysis.

Hepatic lipolysis is mediated by either the classical lipases (*e. g*., ATGL) or autophagy^31, 32^. ATGL activity is inhibited by AMPK, which is a negative regulator of ATGL-mediated lipolysis^35^. Our results showed that neither T_2_ nor T_3_ significantly affected the AMPK–mediated phosphorylation of ATGL so it is likely that the increase in hepatic lipolysis induced by these iodothyronines did not involve stimulation of ATGL activity. Autophagy also has been implicated in the degradation and metabolism of lipids in the liver. In this connection autophagy-deficient mice developed fatty liver disease^32^. Moreover, the transcriptional master regulators of autophagy and lysosomal biogenesis, TFEB and TFE3, also have been linked to lipid catabolism in liver^36^. We previously showed that autophagy-mediated hepatic lipolysis or “lipophagy” was induced by T_3_^26^. In this study, we examined the short-term effects of T_2_ and T_3_ in rats fed HFD and found that autophagic markers such as LC3-II and TFE3 were up-regulated in treated rats when compared to rats fed HFD alone. These findings suggested that lipophagy, rather than stimulation of ATGL, was the major mechanism employed for hepatic TG lipolysis and lipid clearance during the hepatic adaptive phase by these two THs.

Increased rate of FAO often generates cytotoxic free radicals known as reactive oxygen species (ROS) that can damage mitochondria. To sustain mitochondrial function there needs to be efficient mitochondrial quality control that involves co-ordinated mitochondrial biogenesis to maintain a healthy pool of mitochondria as well as anti-oxidant enzymes to get rid of mitochondria damaging free radicals. T_3_ can increase mitochondrial biogenesis^37^, most likely in conjunction with mitophagy^38, 39^. Similarly, we found that T_3_ significantly increased PGC1α, NRF-1, and TFAM in the livers of rats red HFD. In contrast, T_2_ did not increase expression of these proteins. Therefore, although both iodothyronines increased mitochondrial function and FAO, only T_3_ increased mitochondrial biogenesis. In contrast, we found that T_2_ maintained SOD1 protein expression whereas T_3_ decreased its expression in livers from rats fed HFD. Therefore, these results suggest that T_3_ may rely upon mitochondrial turnover whereas T_2_ may relay more on the induction of anti-oxidant proteins such as SOD1 to counteract oxidative stress and maintain quality control of mitochondria.

Sphingolipids are components of membrane bilayers that also serve as regulators for apoptosis, cellular senescence, stress response, inflammation, and metabolism^5^. Ceramides contribute to the cellular damage caused by inflammation from insulin resistance, mitochondrial dysfunction, and oxidative stress in NASH^3^. Currently, little is known about TH effects on ceramide synthesis in hepatic cells. Our results showed that both T_2_ and T_3_, in general, prevented induction of ceramide synthesis across different chain lengths in livers from rats fed HFD. Similarly, sphingomyelin which is associated with fatty liver and insulin resistance^40^, was significantly reduced by both the iodothyronines. Since saturated fats are the precursors of ceramide biosynthesis, the increased FAO in the mitochondria induced by T_2_ and T_3_ may explain the decrease in ceramide and sphingomyelins by these two iodothyronines in livers from rats fed HFD. Our findings have demonstrated a novel role for THs in regulating sphingolipid metabolism and hepatic toxicity through their induction of FAO.

Previously, it was shown that while both T_2_ and T_3_ decreased hepatic steatosis in rats, only T_2_ rescued the insulin resistance associated with fatty liver^13^. HFD inhibits AKT activation to decrease cell survival and increase hepatic metabolic processes such as gluconeogenesis that are negatively regulated by insulin^41^. MAPK/ERK signaling also is critical for insulin signaling in hepatic cells and is down-regulated during NAFLD progression^42^. Since insulin resistance frequently is associated with NAFLD progression, we examined the effects of T_2_ and T_3_ on these two cellular signaling pathways in rats fed short-term with HFD. Interestingly, only T_2_ rescued the negative effects of HFD on AKT and ERK signaling. The inability of T_3_ to increase insulin signaling despite decreasing hepatic fat content shows that there was a dissociation between its lipid-lowering and insulin-sensitizing effects. In agreement with our findings, Vatner *et al*. found that T_3_ paradoxically inhibited insulin signaling in liver despite rescuing fatty liver development in animals fed HFD^43^. In contrast, T_2_ was reported to rescue insulin resistance in NAFLD associated with obesity^13^. The dual effects of T_3_ on lipid metabolism and insulin resistance suggest that the positive effects of T_3_ in reducing liver TG, ceramides, and BCAA are counter-balanced by its potential negative effects on insulin resistance such as generation of diacylglycerol^43^, inhibition of MTORC2^44^, and increased hepatic glucose production. It will be important to determine whether there are dose-dependent effects of T_3_ that preferentially enhance the positive effects on lipid metabolism over the negative ones on gluconeogenesis and diacylglycerol production. In this regard, T_2_ may offer more therapeutic potential than T_3_ since it can increase autophagy and FAO while improving insulin resistance caused by HFD. Our data showing the effects of T_2_ on MAPK/ERK and PI3K pathways also are consistent with those reported by Rochira A *et al*.^45^. According to this study, T_2_ most likely induced these pathways *via* a rapid non-genomic pathway that did not involve TRs^45^. Interestingly we found that that hepatic TRs levels were decreased in animals treated with HFD alone, and with HFD and T_2_ or T_3_. These results support the notion that some of the of the short-term metabolic actions of HFD, T_2_, and T_3_ may not require TRβ1.

In summary, we used a metabolomics approach to obtain a comprehensive and comparative view of the metabolic actions of T_2_ and T_3_ on the livers of rats fed short-term HFD. Both compounds had lipolytic effects in the liver mediated by autophagy and increased FAO although the metabolic profiles suggested that there may be some differences in the mechanism(s) and magnitude of their metabolic effects. It is noteworthy that T_3_ induced mitophagy and mitochondrial biogenesis, whereas T_2_ did not appear to do so despite its ability to induce FAO. Additionally, the increased FAO by both iodothyronines was able to reduce intrahepatic ceramide levels, and thus may protect hepatocytes against lipotoxicity due to increased intracellular saturated fatty acids from intrahepatic hydrolysis of triglycerides and imported FFAs generated by lipolysis of adipose tissue. Last, although there have been several studies showing beneficial effects of both T_2_ and T_3_ for reducing hepatosteatosis^7, 46^, T_2_ may offer potential therapeutic advantages by activating insulin-signaling pathways instead of inducing metabolic and cell signaling effects that counteract insulin action. Although several issues such as the role of TH receptors in T_2_ action and the assessment of the metabolomic changes in chronic HFD models still need to be addressed, our results have provided novel insights into the metabolomic actions of T_2_ and T_3_ during the early hepatic metabolic adaptation to lipid challenge.

## Methods

### Animals and Drugs Treatment

The studies were performed in male Wistar rats (250–300 g) purchased from Charles River Laboratories. They were maintained and used in accordance with the criteria outlined in the Guide for the Care and Use of Laboratory Animals prepared by the National Academy of Sciences and published by the National Institutes of Health. All animals were kept one per cage in a temperature-controlled room at 28 °C under a 12-h light/12-h dark cycle and water was available ad libitum. Rats were divided into four groups and treated for 1 week. The first group (group NCD) received a standard diet (total metabolizable percentage of energy: 60.4 carbohydrates, 29 proteins, 10.6 fat J/J; 15.88 KJ gross energy/g; Muscedola, Milan, Italy). The second (group HFD) received an HFD (consisting of 280 g diet supplemented with 395 g lyophilized lamb meat [Liomellin, Milan, Italy], 120 g cellulose [Sigma-Aldrich, St. Louis,MO], 20 g mineral mix [ICN Biomedical, Solon, OH], 7 g vitamin mix [ICN], and 200 g low-salt butter [Lurpak, Denmark]) (total metabolizable percentage of energy: 21 carbohydrates, 29 proteins, 50 fat J/J; 19.85 KJ gross energy/g). The third group (group HFD-T_2_) received the same HFD together with a daily intraperitoneal injection of T_2_ (25 µg/100 g body wt) (Sigma-Aldrich). The fourth group (group HFD-T_3_) received the same HFD together with a daily intraperitoneal injection of T_3_ (2.5 µg/100 g body wt) (Sigma-Aldrich). After 1 week of treatment, rats were anesthetized by an intraperitoneal injection of chloral hydrate (40 mg/100 g body wt) and then killed by decapitation. Liver was excised and immediately frozen in liquid nitrogen for subsequent analysis. The authors also confirm that all experiments were performed in accordance with relevant guidelines and regulations. The authors confirm that the experimental protocols were approved by Seconda Università degli Studi di Napoli, Caserta, Italy and Duke-NUS Medical School, Singapore institutional committee.

### Metabolomics

Metabolomic analysis was performed as described previously^47^. Amino-acids were extracted from 100 μL of liver homogenate using methanol and then derivatized to form butyl esters using 3 M HCl in butanol. Samples were then reconstituted in 80% aqueous methanol and 4 μL of this solution was injected into an Agilent SB-C8 column (12 × 50 vmm with 1.8 um particle size) for analysis. Mobile phase used was 80% methanol and 20% water, and flow rate was maintained at 0.4 ml/min for 2 min. Isocratic flow of 0.6 ml/min of 30% acetonitrile and 70% water with 0.1% formic acid was maintained for 5.5 min. For Sphingolipid analysis tissue homogenate was resuspended in 900 μl of ice-cold chloroform-methanol (1:2) and incubated in ice for 15 min with vortexing every 5 min. Three hundred microliters of ice-cold distilled water (dH2O) and 300 μl of ice-cold chloroform were added to the samples, which were then vortexed and centrifuged at 8,000 × g for 2 min at 4 °C. The lower organic phase was transferred into a clean microcentrifuge tube. A second extraction was performed by adding 300 μl of ice-cold chloroform, and the lower organic phase was pooled with that of the first extraction. For acylcarnitine extraction, 100 µL of tissue homogenate was extracted using methanol. The acylcarnitine extracts were derivatised with 3 M Hydrochloric acid in methanol, dried and reconstituted in methanol for analysis in LC-MS.The collected samples were dried under a stream of nitrogen and stored at −80 °C until ready for liquid chromatography tandem mass spectrometry (LC-MS/MS) analysis. Data acquisition and analysis were performed on Agilent MassHunter Workstation B.06.00 Software.

### Western blotting

Tissue samples were lysed using CelLytic™ M Cell Lysis Reagent (Sigma, C2978) and immunobloting was performed as described previously.25 Image acquisition was done using ChemiDoc (Bio-Rad ChemiDoc™ MP System, 1708280). Densitometry analysis was performed using ImageJ software (NIH, Bethesda, MD, USA).

### Reagents

Antibody details are as follows: TFAM (Cell Signaling Technology, 7495); PGC-1α (Santa Cruz Biotechnology, sc-13067); NRF1 (Cell Signaling Technology, 12381); UCP2 (Santa Cruz Biotechnology, sc-6525); CPT1α (Abcam, ab128568); phoshpo-Acetyl-CoA Carboxylase (Cell Signaling Technology, 11818); Acetyl-CoA Carboxylase (Cell Signaling Technology, 4190S); LC3B-II (Cell Signaling Technology, 2775); TFE3 (Cell Signaling Technology, 14779); TFEB (Abcam, ab2636); phospho-ATGL S406 (Abcam ab135093); ATGL (Abcam ab57562); Phospho-p44/42 MAPK (Erk1/2) Thr202/Tyr204 (Cell Signaling Technology, 9101); p44/42 MAPK (Erk1/2) (Cell Signaling Technology, 9102); Phospho-Akt S473 (Cell Signaling Technology, 4058); Akt (Cell Signaling Technology, 9272); SOD1 (Cell Signaling Technology, 4266); ACTB/β-Actin (Santa Cruz Biotechnology, sc-81178); GAPDH (Cell Signaling Technology, 2118).

### Liver triglyceride estimation

Liver triglycerides were estimated using manufacturer’s guidelines (Cayman chemicals, Item No: 10010303)

### Statistical analysis

Results are expressed as means (n = 4 ± SEM). The statistical significance of differences between groups was determined using one-way ANOVA followed by a Student-Newman-Keuls test. Differences were considered significant at P < 0.05.

## Electronic supplementary material

Supplementary Figures and legends
