# Supplementary material for: Metabolomic analysis shows differential hepatic effects of T2 and T3 in rats after short-term feeding with high fat diet
Source: Sci Rep. 2017 May 17;7:2023. doi: 10.1038/s41598-017-02205-1 (PMC5435676; doi:10.1038/s41598-017-02205-1)
Supplement: Supplementary file 1 — Supplementary Figures and legends [file 41598_2017_2205_MOESM1_ESM.pdf]

Metabolomic analysis shows differential hepatic effects of T_2 and T_3 in rats after short-term feeding with high fat diet

Liliana F. Iannucci[1,3], Federica Cioffi[2], Rosalba Senese[3], Fernando Goglia[2], Antonia Lanni[3,*], Paul M. Yen[1,*], Rohit A. Sinha[1,*]

Supplementary figure legends

Supplementary Figure 1. Animals body weight, blood glucose T_3 and T_4 levels. The table shows the animals body weight, circulating levels of T_3 and T_4 and glucose (fasting) after 1 week of diet regime. Values are means ±SEM (n=4). *P <0.05 in NCD Vs HFD; $^{\#}$P <0.05 in HFD Vs HFD+T_2/HFD+T_3.

Supplementary Figure 2. Hepatic aminoacids and organic acids metabolism are regulated by T_2 and T_3 in HFD fed rats. Aminoacids concentration measured in hepatic rat tissues. Values are means ±SEM (n=4). *P <0.05 in NCD Vs HFD; $^{\#}$P <0.05 in HFD Vs HFD+T_2/HFD+T_3.

Supplementary Figure 3. Differential THs effects on mitochondrial biogenesis. Representative Immunoblot and densitometry showing proteins content of mitochondrial biogenesis **(A-B)** and mitochondrial dynamics markers **(C-D)**. Values are means ±SEM (n=4). *P <0.05 in NCD Vs HFD; $^{\#}$P <0.05 in HFD Vs HFD+T_2/HFD+T_3.

Supplementary Figure 4. HFD-induced SOD1 expression is prevented by T_3 but not T_2 in rat livers. Representative Immunoblot and densitometry showing proteins content of the antioxidant enzyme SOD1. Values are means ±SEM (n=4). *P <0.05 in NCD Vs HFD; #P <0.05 in HFD Vs HFD+T_2/HFD+T_3.

Supplementary Figure 5. HFD-reduced TRβ1 levels in rat livers. Representative Immunoblot and densitometry showing proteins content of TRβ1. Values are means ±SEM (n=4). *P <0.05 in NCD Vs HFD; $^{\#}$P <0.05 in HFD Vs HFD+T_2/HFD+T_3.

	Body weight (g)	TT4 (nM)	TT3 (nM)	Glucose (mg/dl)
NCD	320 ± 10	62 ± 3,8	0,79 ± 0,04	82 ± 2,2
HFD	340 ± 11	71 ± 4,9	0,82 ± 0,05	94,1 ± 3,8
HFD-T_2	325 ± 9	67 ± 3,2	0,76 ± 0,03	87 ± 3,2
HFD-T_3	322 ± 8	58 ± 2,1	1,45 ± 0,12*	88 ± 3,4

*$P < 0.05$ vs. NCD, HFD, HFD-T_2.

Suppl Figure 1

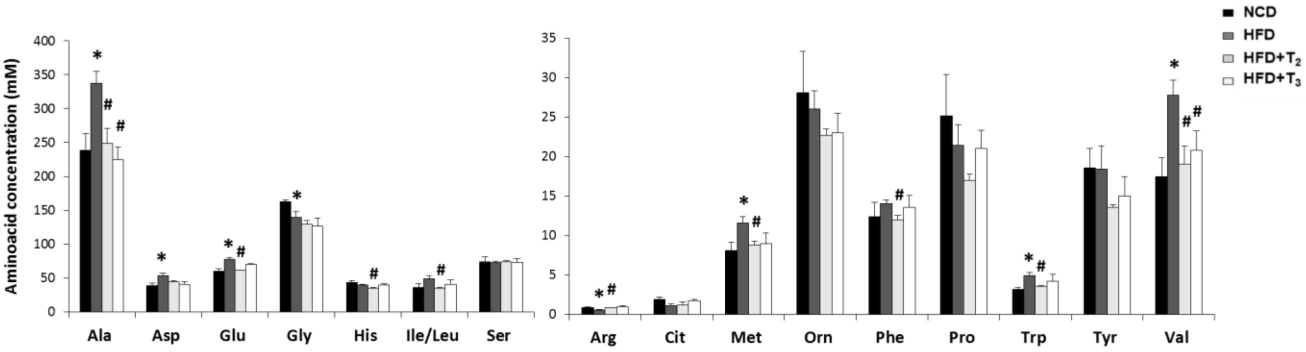

Suppl Figure 2

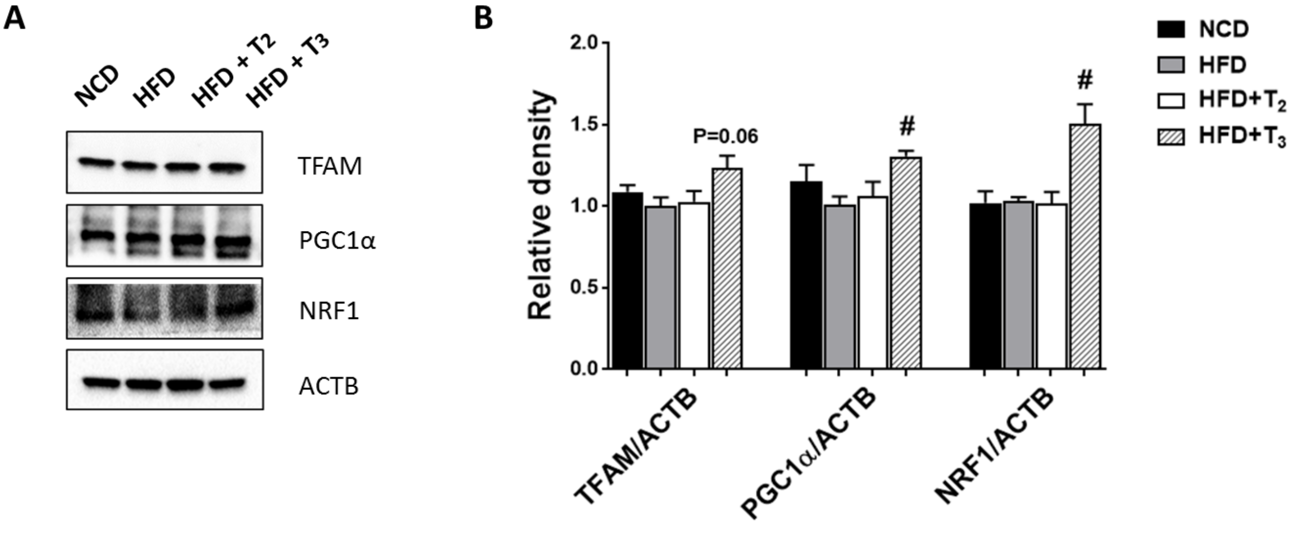

Suppl Figure 3

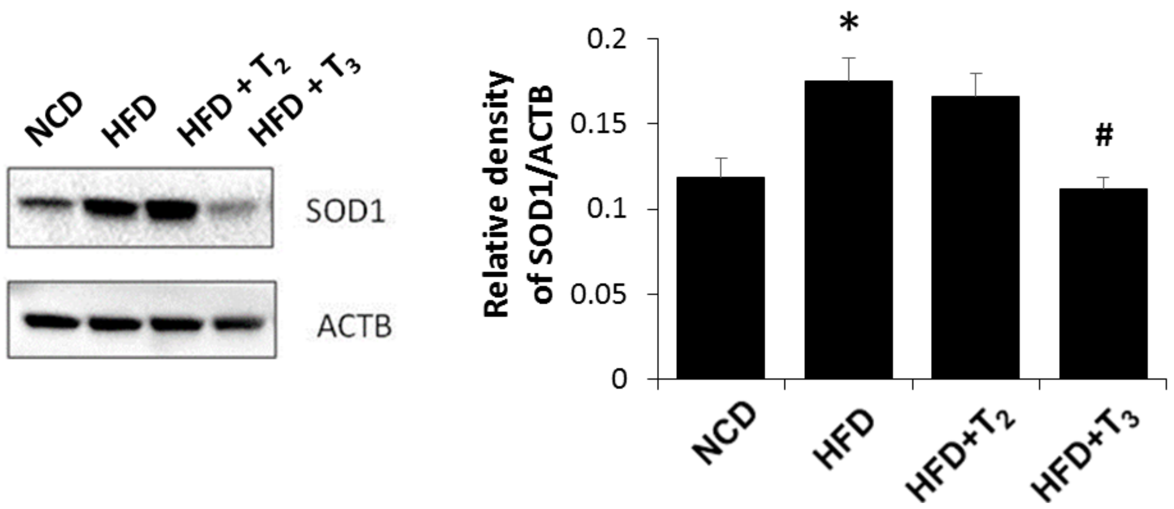

Suppl Figure 4

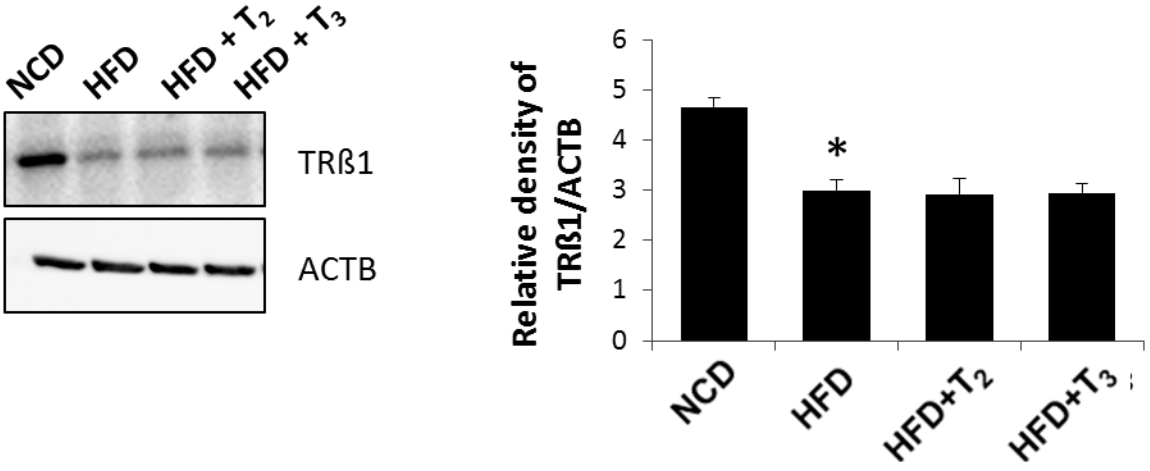

Suppl Figure 5
